# Understanding homeowner decision-making through demographics: Barriers to housing retrofit at scale

**DOI:** 10.1371/journal.pone.0338740

**Published:** 2025-12-29

**Authors:** Chamara Panakaduwa, Paul Coates, Mustapha Munir

**Affiliations:** School of Science, Engineering & Environment, University of Salford, Crescent, Salford, United Kingdom; Hunan University, CHINA

## Abstract

Housing retrofit is vital for meeting sustainability goals, yet limited homeowner demand remains a significant barrier to widespread adoption. While challenges such as skills shortages and supply chain issues also exist, a strong homeowner demand could drive solutions to these obstacles. Despite the availability of grant schemes, homeowner interest in retrofitting remains low, slowing adoption rates and impeding national sustainability targets. Based on data from a questionnaire survey of UK homeowners, this study explores how demographic factors shape homeowner decision-making behaviour. Statistical analysis reveals several key insights: (1) homeowners primarily view retrofitting as an investment, seeking returns through energy bill savings; (2) concern over unintended consequences of retrofitting impacts willingness to participate; and (3) educational level strongly influences homeowner behaviour. These findings offer valuable perspectives for developing large-scale retrofit strategies, addressing the underlying behavioural barriers that have hindered the success of existing retrofit programs.

## 1. Introduction

The energy used in the building sector is reported to contribute 17.5% to global carbon emissions [1]. In the context of the United Kingdom, the building sector contributes 27% to carbon emissions. This contribution is shared by residential buildings at 18%, commercial buildings at 6% and public buildings at 3% [2]. Although the UK has reduced their emissions by 52.7% from 1990 to 2023, the progress related to residential building decarbonisation is only 35.1%. The world is going to achieve net-zero targets, and the UK Government is legally bound to reach net zero by 2050 [3]. Considering these aspects, it is time to minimise carbon emissions from residential stock. Energy efficiency retrofit is the required action to make the residential stock future-proof with carbon reduction goals.

Although the International Standards Organisation does not define retrofit directly, the idea of retrofit can be identified as “Modification to an asset to generate an improved condition” [4]. The British Standard Organisation identifies residential retrofit as installing measures in an existing house to improve energy efficiency and ventilation or reduce carbon emissions. Although the main focus of retrofit is observed to be related to energy efficiency, there are other benefits which are not subordinate. Some examples are improving health and well-being, enhancing occupant comfort, reducing environmental impact and preserving heritage aspects [5].

Driving housing retrofit has been defined as a tripartite game among the government, the homeowners and the contractors. The government is to provide incentives and supervision (regulation), contractors to supply retrofit measures (supply side) and homeowners to adopt retrofit measures (demand side) [6]. Another study highlighted the importance of a balance between reducing energy consumption and reducing emissions, as well as occupant health, comfort, and architectural heritage [7]. Considering these aspects, the PAS 2035:2023 specification has highlighted the purpose of the retrofit standards: to reduce the risks to both the occupants and the dwelling [5]. PAS 2035 specification can be considered the voluntary standard for housing retrofit in the UK, although it is mandatory for government-funded retrofit projects.

Retrofit is ideally viewed from a multi-stakeholder perspective where everyone is happy. It is all about making an old building more sustainable, comfortable, enjoyable, valuable, energy efficient, aesthetic, and cheaper to use [8]. The homeowner can be identified as the most prominent stakeholder in driving energy retrofits [9]. First, they own most of the residential buildings. 63.1% of the house tenures are owner-occupiers, and a further 19.1% of house tenures are private rented [10]. Second, they are the ones who ultimately decide whether to invest in retrofit measures or not, as they are responsible for the cost and implementation of such upgrades [11]. The poor trust and lack of awareness about housing retrofit have diminished homeowners’ demand for housing retrofit [12].

The justification for retrofit is already there. The problem is that the homeowners do not find it, as people do not make decisions rationally, as suggested by Lutzenhiser [13]. Although the benefits of retrofitting houses are explicitly conveyed, people hesitate to make positive decisions to retrofit their homes. Difficulties in convincing homeowners to retrofit and the lack of business models to support sustainability are key challenges in driving retrofit. Further, one of the main challenges in driving retrofit is to change the focus to the retrofit. Paying for housing retrofit is viewed under energy bill savings and investment value creation. The health and environmental benefits of retrofit are often ignored [14].

One of the severe criticisms of housing retrofit policymaking is the overreliance on technical rational models. Several retrofit initiatives were reported to fail as they have only focused on the rational decision-making of the homeowners [15–17]. According to Hoffeld, emotions play a key role in the decision-making of people. There is a famous saying that “People buy on emotions and justify with logic” [18]. Further, heuristics also play a critical role in non-rational decision-making according to Todd & Gigerenzer [19]. It is not expected to go into detail about the decision-making processes, as the focus is on the demographic influence on the homeowners’ retrofit decision-making process.

Nanda highlights the problem of unintended consequences due to the poor installation of retrofit measures. This is due to the poor workmanship of the installers [20]. Because of the increased criticisms of the quality of retrofit projects, the Each Home Counts Review was commissioned by the UK government and was published in 2016 [5,21]. The recommendations were taken into consideration for the adoption of the PAS 2035 framework to provide clear processes to minimise risks, ensure retrofit is professional work, provide explicit definitions of intended outcomes with responsibility and ensure clients’ confidence [5,22,23].

Technology, finance, supply chain, workforce, and management are problems that drive retrofit at the national level. Even if all the other challenges are addressed, housing retrofit will not happen without the consent of the homeowner [24]. The most problematic challenge can be suggested as the limited demand from homeowners to retrofit their houses. It can be argued why the government cannot make retrofitting houses a legal requirement. The answer is that the people can change the government. This is the reason that pushed the UK government to compromise on Minimum Energy Efficiency Standards (MEES) laws, mandatory gas boiler phase-out, and mandatory fossil fuel vehicle phase-out [25]. The legal provisions may be used for the laggards segment of homeowners, when 84% of the housing stock is retrofitted according to the diffusion of innovations theory [26].

Several researchers have studied the decision-making behaviour of homeowners. Ebrahimigharehbaghi et al have found that the homeowners are more likely to make decisions which cannot be explained through rational perspectives [27]. Liu et al have found that younger homeowners are more likely to see retrofitting houses positively [28]. Lack of awareness has been identified as a key reason for homeowners to engage in retrofit [29]. Most of the studies are related to the general factors influencing homeowner decision-making in retrofitting. The influence of demographic factors on the retrofit decision-making behaviour of the homeowners was less studied. A research gap is how demographic factors influence homeowner decision-making behaviour in housing retrofit. Soft measures need to be taken to promote housing retrofit by winning the homeowners’ hearts, not by forcing them on them. It is important to understand the homeowners’ retrofit decision-making behaviour and strategically plan the delivery of housing retrofit accordingly. This study was conducted with the research question of how the demographics of the homeowners influence their decision-making behaviour in housing retrofit.

## 2. Materials and methods

This study used a quantitative data collection approach, using a questionnaire survey. Primary data was collected from UK homeowners under the ethical approval reference number 10162 dated 09^th^ May 2023 from the University of Salford, United Kingdom. The questionnaire was randomly distributed by sharing an invitation letter and the participant information sheet with a QR code for the survey. Further, the invitation was shared in online community groups in the UK. The data was collected from the respondents during the period from 16^th^ July 2023–22nd August 2023. All the respondents were adults, and their written informed consent was recorded online. The respondents who provided informed consent for the study were facilitated to proceed with the questionnaire survey.

The summary of the methodology is given in Table 1. In this questionnaire survey, data were collected from a sample of 104 homeowners in the UK. Focusing on the UK context, there are around 23.5 million households under owner-occupier and privately rented tenures [10]. The sample size was calculated using Morgan’s table with a 90% confidence interval and a 10% margin of error. This led to an expected sample size of 69. Considering the nature of studies and the level of precision required, this level of confidence and margin of error were adequate.

**Table 1:** Methodology summary.

Item	Description
Type of data collection	Questionnaire survey
Purpose	To understand the homeowner’s decision-making behaviour
Type of data	Quantitative primary data
Questionnaire codes	1. Cost, 2. Finance, 3. Grants, 4. Time, 5. Quality, 6. Performance, 7. Disruptions, 8. Options, 9. Stakeholders, 10. Risk.
Number of questions	Five questions for each code
Nature of data	Five-point Likert scale data
Sample	The UK homeowners
Sampling method	Simple random sampling
Sample size	Expected 70, collected 104
Time scale	July & August 2023

**Fig 1:**
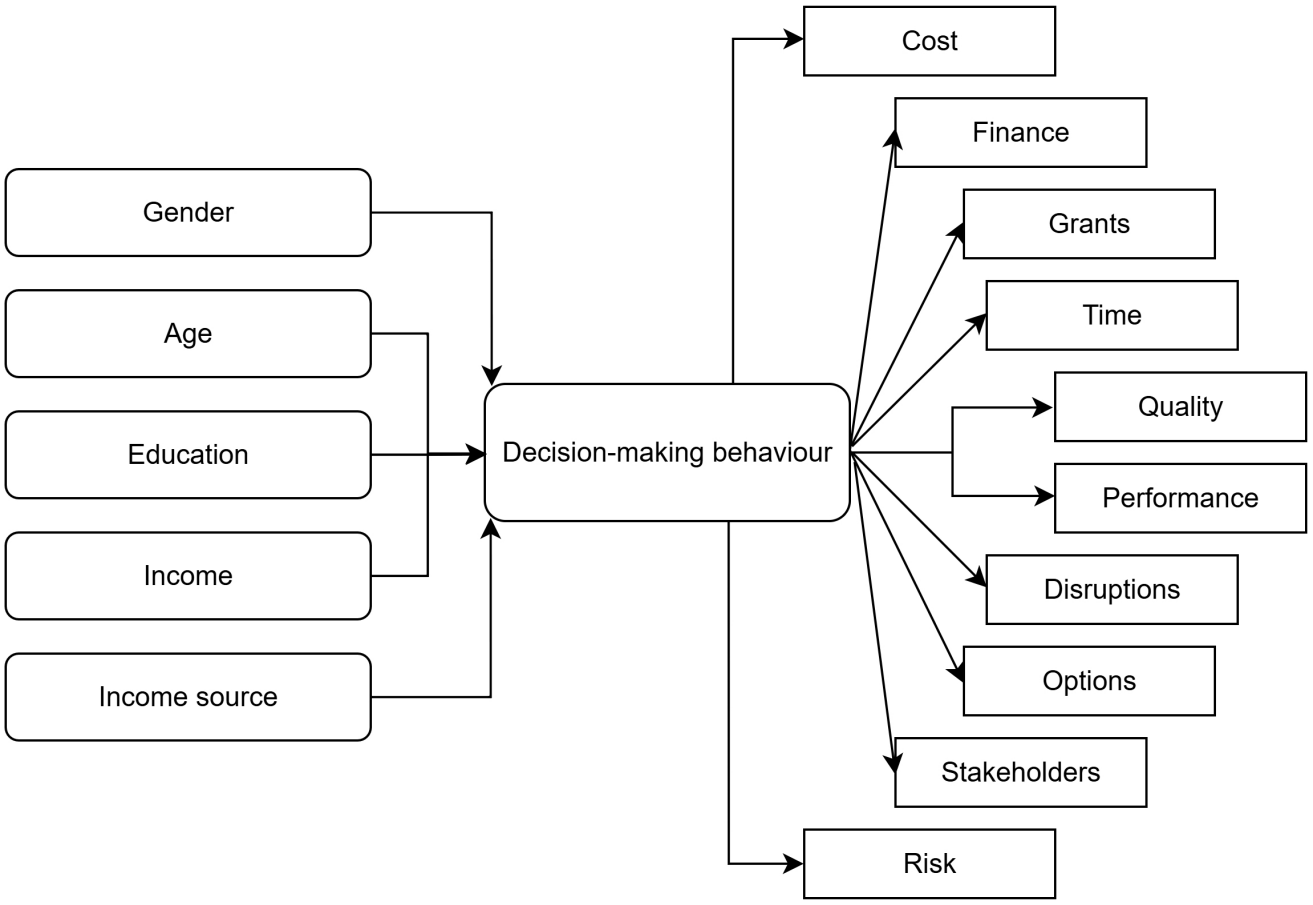
Conceptual framework.

Fig 1 shows the conceptual framework used for this study. First, the demographic details of the homeowners were collected. Second, ten different information factors were presented to the homeowners, and they were asked how they would value these information factors when making a retrofit decision. Fifty potential information factors under ten codes were presented to the respondents.

Table 2 shows which statistical tests were used to achieve different study objectives. An Exploratory Factor Analysis (EFA) was performed first to assess the construct validity of the dataset. A Principal Axis Factoring was conducted on each of the five items representing the ten codes of the questionnaire. Above 0.7 Kaiser–Meyer–Olkin (KMO) measure of sampling adequacy indicates higher suitability for factor analysis. Bartlett’s Test of Sphericity was significant (p < .001) for all the codes, and this confirmed a sufficient inter-item correlation for the justification of factor extraction.

**Table 2:** Study objectives and statistical tests.

	Objectives	Statistical test
1	Sampling adequacy	Kaiser-Meyer-Olkin (KMO)
2	Sufficiency of inter-item correlations	Bartlett’s Test of Sphericity
3	Construct validity	Exploratory Factor Analysis (EFA)
4	Internally consistency	Cronbach’s Alpha
5	Mean values to identify the most important information factors.	Descriptive statistics
6	Homogeneity of variances of the sample	Levene’s Test of Equality of Error Variances
7	To identify how different demographic clusters value different information factors.	Sectorial analysis of variance (ANOVA) Standard ANOVA (equal variances) Welch’s ANOVA (unequal variances)
8	Post hoc tests	Tukey if variances equal and Games–Howell if variances unequal.

All five items for each code supported the construct’s convergent validity with 92% of items having above 0.7. Rotation was not applied since only one factor was retained. The results confirmed that the items collectively represent a single latent construct with strong internal coherence. Cronbach’s alpha for each five items was above 0.7, indicating acceptable internal consistency reliability. These results satisfactorily demonstrated that the items represent an internally consistent construct, ensuring construct validity of the dataset. Five-point Likert-type questions were used to identify the most important information factors. The general mean values were used for the purpose. Items with mean values above 3.9 were considered the most important information factors. The scale was from 1 to 5 (1 being not at all important and 5 being extremely important).

Levene’s Test of Equality of Error Variances was conducted to understand the homogeneity of variances of the sample. Either standard ANOVA or Welch’s ANOVA was performed according to Levene’s test. Post-hoc tests were conducted after finding statistically significant results with the ANOVA tests for categorical variables with three or more levels (income source). The post hoc tests are to understand where exactly the significant difference is. Full details related to the calculations can be found in this data repository [30].

According to Kothari, Likert scale data is ordinal, and the distance between two consecutive numbers may not be similar [31]. Meanwhile, some authors argue that there is no problem with treating Likert data as interval data [32]. Boone & Boone argue that when several Likert questions are testing the same attitude or trait, the Likert data behaves highly similarly to interval data, and there is no issue with using statistical tests measuring the central tendency [33]. E.g., Mean, mode, median or standard deviation. This study uses five questions to test one construct (code). Creswell & Creswell state that when the Likert question has five or more categories of responses, Likert scale data behave similarly to interval data [34]. This study has used five such categories of responses (five-point Likert style).

The overall purpose of this study is to generally understand the homeowner decision-making behaviour in housing retrofit. This was approached by understanding their information requirements for retrofit decision-making. Accordingly, these information requirements need to be ranked and prioritised. The analysis does not need to be 100% precise as the analysis is already constrained by bounded rationality. Considering these factors, the above statistical tests were used despite the limitation of assuming Likert data as interval data.

## 3. Results

### 3.1. Demographics

The survey was responded to by 104 homeowners. The minimum required number of responses was 69 to achieve a 90% confidence interval and 10% margin of error. As the purpose was to understand decision-making behaviour, the focus was on the respondent’s background, not on the house demographics. How respondents from different demographics value different information (to make a retrofit decision) was studied. Considering the respondents’ fatigue and bounded rationality concerns, the following five demographic factors were collected: gender, age group, education, income source, and income level.

**Fig 2:**
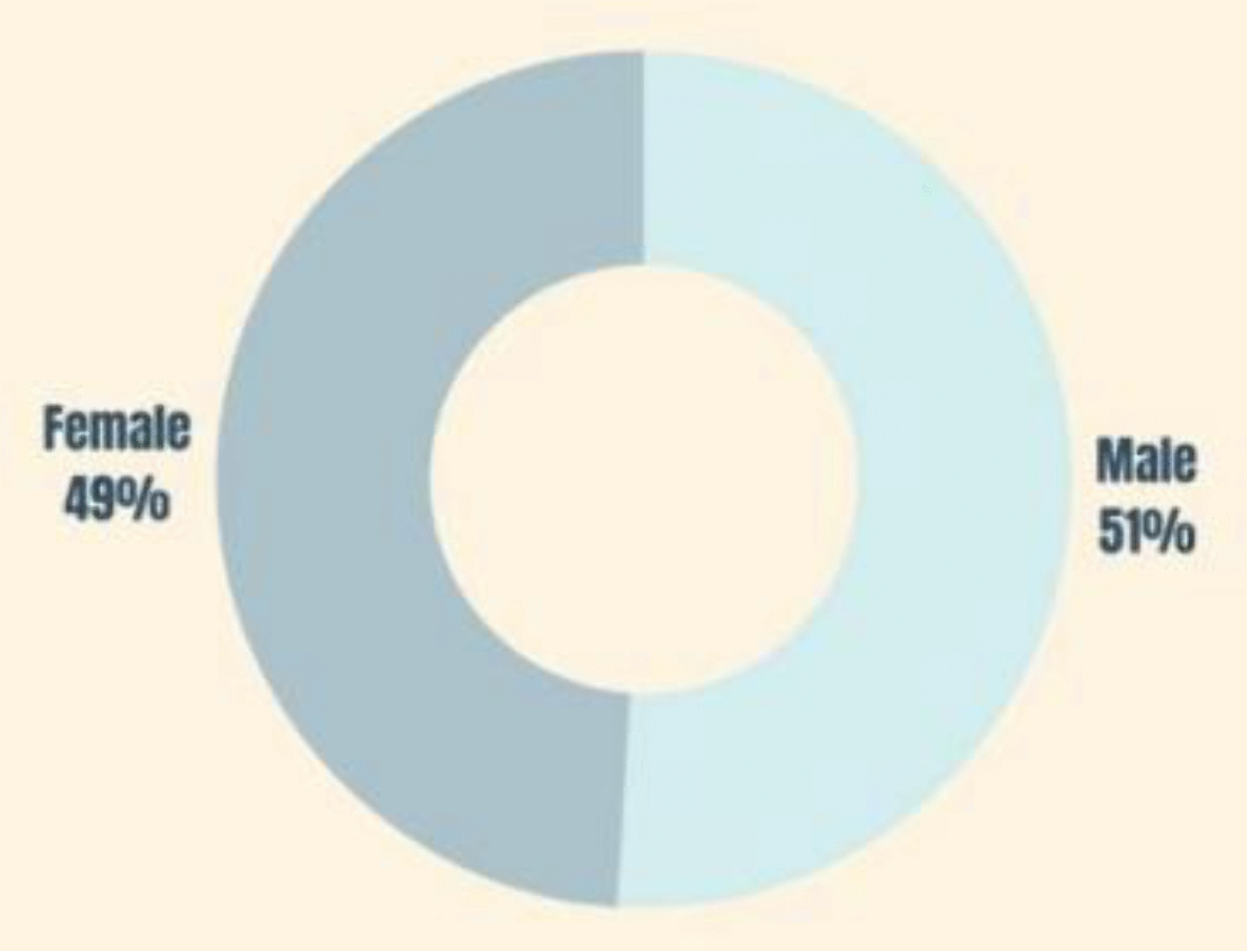
Gender distribution of the sample.

According to Fig 2, the sample is representative of gender. Both male and female respondents responded to the questionnaire in a similar way.

**Fig 3:**
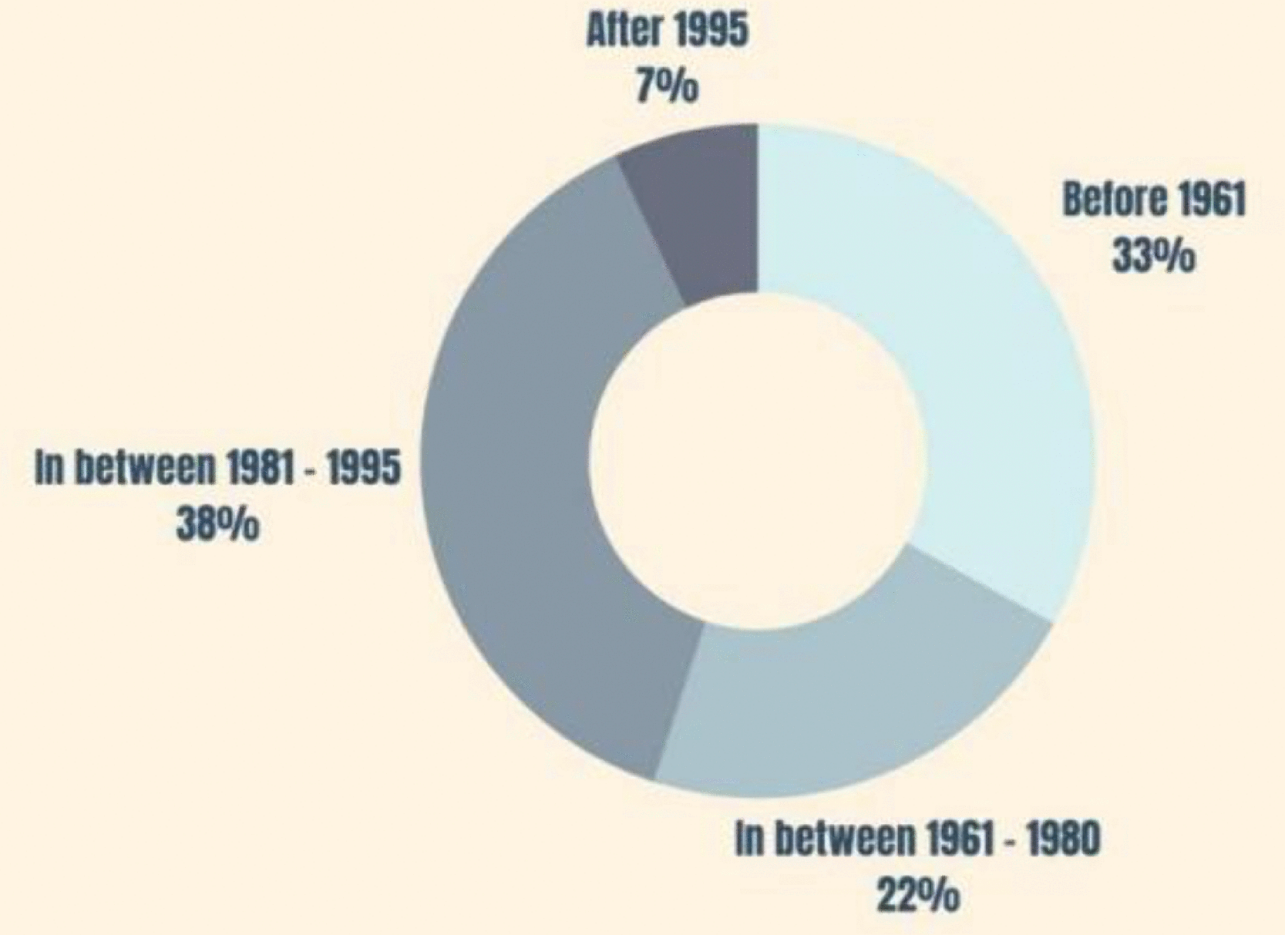
Age group distribution of the sample.

According to Fig 3, the age groups of the sample were collected to separate the respondents into different generations according to the generations theory [35]. Respondents born before 1961 belong to older generations, such as baby boomers or the silent generation. Accordingly, this study was responded to by people from older generations, Generation X, Generation Y, and Generation Alpha. Statistical tests were conducted to see whether there are any differences among these generations in retrofitting decision-making information factors.

**Fig 4:**
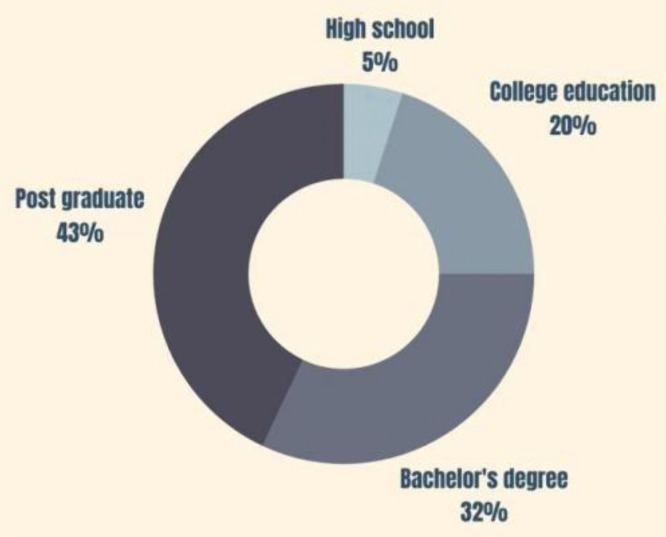
Education distribution of the sample.

According to Fig 4, other than the 5% of respondents who only have a high school education, 20% of the respondents have a college education, and the balance, 75%, are degree holders. Considering the above, it can be concluded that the sample is highly educated, which is also a limitation.

**Fig 5:**
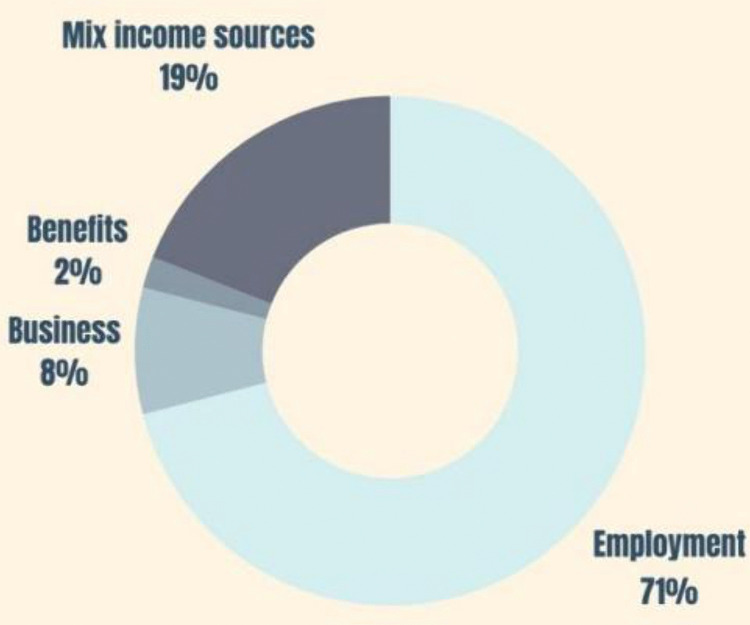
Income source distribution of the sample.

According to Fig 5, most of the sample (71%) is employed. 8% of the sample is engaged in businesses, while 19% of the sample has multiple sources of income. By considering this, it can be expected that the sample mainly represents the view of employed homeowners. This is a limitation of the study.

**Fig 6:**
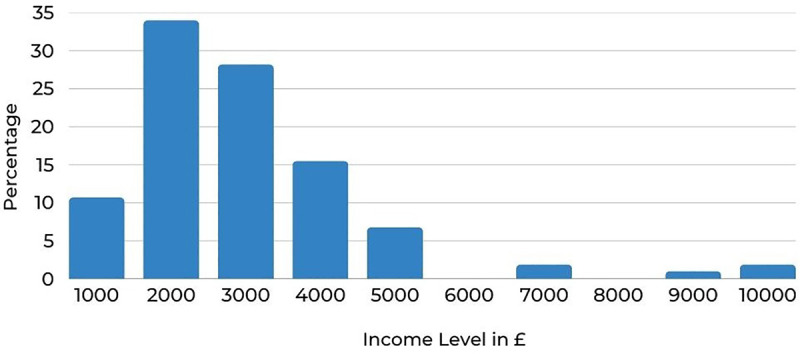
Income level distribution of the sample.

According to Fig 6, income is distributed from £1,000 per household to £5,000. The curve is skewed to the right. There are a few outliers for income levels of £7,000, £9,000 and £10,000 per month. Considering the above distribution of income levels, it can be suggested that the statistical test results involving income levels are statistically significant.

### 3.2. Frequency analysis

#### 3.2.1. Construct validity and internal consistency.

Table 3 shows the results of the tests conducted for sampling adequacy. Ranging from 0.752 to 0.884, meritorious levels were achieved for all variables under the Kaiser–Meyer–Olkin (KMO) measure. Statistically significant results were achieved with Bartlett’s Test of Sphericity for all ten sets of items. This confirms the sufficient inter-item correlation for the factor extraction.

**Table 3:** Sampling adequacy.

	Q1 (Cost)	Q2 (Finance)	Q3 (Grants)	Q4 (Time)	Q5 (Quality)	Q6 (Performance)	Q7 (Disruption)	Q8 (Options)	Q9 (Stakeholders)	Q10 (Risk)
KMO	.872	.884	.870	.785	.853	.752	.874	.867	.863	.856
Bartlett’s (df)	10	10	10	10	10	10	10	10	10	10
Bartlett’s (Sig.)	<.001	<.001	<.001	<.001	<.001	<.001	<.001	<.001	<.001	<.001

Table 4 shows the Exploratory Factor Analysis (EFA) using Principal Axis Factoring for all the codes of the dataset. The results show that 92% of the factors have over 0.7 loading, corresponding to a higher internal consistency and unidimensionality of the scale. This indicates that all items measure the same underlying concept.

**Table 4:** Exploratory Factor Analysis (EFA).

	Q1 (Cost)	Q2 (Finance)	Q3 (Grants)	Q4 (Time)	Q5 (Quality)	Q6 (Performance)	Q7 (Disruption)	Q8 (Options)	Q9 (Stakeholders)	Q10 (Risk)
QX.1	.874	.969	.917	.872	.908	.852	.890	.920	.927	.829
QX.2	.871	.963	.865	.834	.907	.828	.855	.894	.863	.822
QX.3	.838	.944	.863	.790	.895	.792	.846	.889	.853	.803
QX.4	.837	.827	.814	.780	.790	.759	.784	.880	.835	.775
QX.5	.805	.625	.716	.684	.781	.689	.769	.822	.808	.678

Table 5 shows the internal consistency measures of the questionnaire codes under the Cronbach’s Alpha test. According to George & Mallery [36], internal consistency is considered acceptable when the Cronbach’s Alpha values are higher than 0.7 (It is good over 0.8 and excellent over 0.9). According to Table 5, all the questionnaire codes have a value above 0.8. It was concluded that all the questions in the questionnaire measured the same construct under their respective codes. For example, all five questions under the code “Cost” measure cost factors, and they are internally consistent.

**Table 5:** Cronbach’s Alpha test for questionnaire codes.

	Q1 (Cost)	Q2 (Finance)	3 (Grants)	Q4 (Time)	Q5 (Quality)	Q6 (Performance)	Q7 (Disruption)	Q8 (Options)	Q9 (Stakeholders)	Q10 (Risk)
Cronbach’s Alpha	0.883	0.932	0.945	0.917	0.886	0.931	0.892	0.919	0.938	0.925
Number of items	5	5	5	5	5	5	5	5	5	5

#### 3.2.2. Data analysis for the codes.

The mean values of the items are used to rank the items in a code. There are ten codes in this study, and there are five items per code. The purpose of this analysis is to study the behaviour of homeowners by looking at how they value information related to housing retrofit. One limitation of the analysis is treating ordinal Likert-type data as interval data when the frequency analysis was done. In this regard, a deeper evaluation of the frequency analysis is not expected. The objective is to identify the behaviour. According to the questionnaire coding, level 1 refers to the response “Not at all likely” and the level 5 refers to “Extremely likely”. There are three incremental levels in between: 2 – Less likely, 3 – Somewhat likely, 4 – A lot likely. Considering the overall results, over 3.9 ranked responses were considered as more important.

Table 6 shows the twenty most prominent information factors in the order of diminishing priority, according to the respondents’ collective opinions. The questionnaire contained 50 questions containing information factors related to housing retrofit decision-making under ten codes. Mean value was used to determine the importance of the information factors.

**Table 6:** Frequency analysis for the questionnaire codes.

Rank	Information factor	Mean value	Median value	Mode	Std. Deviation
1	Estimated energy use reductions.	4.2500	5.00	5.00	0.9729
2	Estimated future energy bills.	4.2500	5.00	5.00	1.0120
3	Warranty and guarantee information about upgrade measures.	4.2255	5.00	5.00	1.0331
4	Comparison of future and current energy bill/use reductions.	4.2115	5.00	5.00	0.9821
5	Quality ratings of the suppliers, installers, designers and others.	4.1250	4.00	5.00	0.9822
6	Monthly loan instalments and payback period.	4.0769	5.00	5.00	1.1716
7	Overall upgrade project duration.	4.0481	4.00	5.00	1.0463
8	Recommended home upgrade measures.	4.0388	4.00	5.00	1.0839
9	Health and safety risks.	4.0388	4.00	5.00	0.9591
10	The big picture of home upgrades.	4.0192	4.00	5.00	1.0701
11	Possibility of breaking the project into phases and their costs.	4.0097	4.00	5.00	1.0891
12	Cost of each housing upgrade activity.	3.9709	4.00	5.00	1.1329
13	Guaranteed minimum quality of upgrade measures.	3.9515	4.00	5.00	0.9938
14	Potential risk towards cost, time, and quality.	3.9327	4.00	5.00	0.9580
15	Types and amounts of finance.	3.9327	4.00	5.00	1.2248
16	Potential renewable energy generation by the house.	3.9320	4.00	5.00	1.0503
17	Nature of disruption.	3.9231	4.00	5.00	1.0305
18	Quality information about the products and materials.	3.9135	4.00	5.00	1.1330
19	The total upfront cost of the housing upgrade.	3.9135	4.00	4.00	1.3080
20	Cost comparisons among sources/contractors.	3.9126	4.00	5.00	1.0946

### 3.3. Analysis of variance

ANOVA test is a parametric test that relies on the critical assumption about the homogeneity of Variances. In order to understand the homogeneity of the sample, a Levene’s Test of Equality of Error Variances was conducted first.

Table 7 shows the results of Levene’s test. The assumption of homogeneity of error variances was met with the seven codes: cost, grants, time, performance, disruption, stakeholders and risk. The standard ANOVA test was conducted for these codes. Welch’s ANOVA test was conducted for finance, quality and options codes, as they did not meet the assumption of homogeneity of variances. In order to avoid confusion and make the table clearer, only the statistically significant results are reported.

**Table 7:** Levene’s test of equality of error variances.

	Q1 (Cost)	Q2 (Finance)	3 (Grants)	Q4 (Time)	Q5 (Quality)	Q6 (Performance)	Q7 (Disruption)	Q8 (Options)	Q9 (Stakeholders)	Q10 (Risk)
df	(6,95)	(6,96)	(6,93)	(6,96)	(6,92)	(6,95)	(6,93)	(6,94)	(6,95)	(6,92)
F	2.009	2.898	.437	2.198	2.637	1.556	2.146	3.549	1.695	1.223
p	.072	.012	.853	.050	.021	.169	.055	.003	.131	.302

Table 8 shows a summary of the results of the ANOVA tests. This ANOVA test aims to understand decision-making behaviour by looking at how homeowners from different demographic sectors seek information to make a retrofit decision. Five demographic variables are considered in this study: gender, age group, education, income source and income level. In this analysis, the demographic sectors are the independent variables, and the information factors are the dependent variables. Statistics show the comparative value of the influence, while the significance shows the possibility of having the result by chance. Partial eta square is a measure of effect size, indicating the practical significance. It communicates the real-world significance of the relationship between independent and dependent variables.

**Table 8:** ANOVA summary table for homeowner demographics.

	Description	D1 Gender	D2 Age group	D3 Education	D4 Income source	D5 Income level
Cost (Std. ANOVA)	F	–	–	7.319		–
	Significance	–	–	.008		–
	df			(1,92)		
	Partial ŋ^2^			0.074		
Finance (Welch’s ANOVA)	F	–	5.493	–	–	–
	Significance	–	.002	–	–	–
	df		(3,100)			
	Partial ŋ^2^		.141			
Grant (Std. ANOVA)	F		4.135	4.538		
	Significance		.045	.036		
	df		(1,90)	(1,90)		
	Partial ŋ^2^		.044	0.48		
Time (Std. ANOVA)	F	–	–	5.517	–	–
	Significance	–	–	.021	–	–
	df			(1,93)		
	Partial ŋ^2^			0.56		
Quality (Welch’s ANOVA)	F	–	–	–	–	–
	Significance	–	–	–	–	–
	df					
	Partial ŋ^2^					
Performance (Std. ANOVA)	F	–	–	9.738	–	–
	Significance	–	–	.002	–	–
	df			(1,92)		
	Partial ŋ^2^			.096		
Disruptions (Std. ANOVA)	F	–	–	6.698	–	–
	Significance	–	–	.011	–	–
	df			(1,90)		
	Partial ŋ^2^			.069		
Options (Welch’s ANOVA)	F	–		3.848	–	–
	Significance	–		.012	–	–
	df			(3,98)		
	Partial ŋ^2^			.105		
Stakeholders (Std. ANOVA)	F	–	–	10.182	–	–
	Significance	–	–	.002	–	–
	df			(1,92)		
	Partial ŋ^2^			.100		
Risk (Std. ANOVA)	F	–	–	5.325	–	–
	Significance	–	–	.023	–	–
	df			(1,89)		
	Partial ŋ^2^			.056		

The above results show the existence of a relationship between the variables (F) and the importance of the relationship (Partial ŋ^2)^. Accordingly, there is a relationship between education and all the information factors except finance and quality. Further, a relationship was found between the age group variable and the finance and quality variables. These results do not show the direction of relationship. The direction of the relationship is not applicable to nominal independent variables; gender and income source. B Coefficient (Regression Coefficient) was used to understand the direction of the relationship.

Table 9 shows the B coefficients for the non-categorical variables (age group, education and income level). Only the statistically significant results are reported. It can be observed that significant results are only available for the independent variable “Education”.

**Table 9:** B Coefficient for non-categorical variables.

		Q1 (Cost)	Q2 (Finance)	3 (Grants)	Q4 (Time)	Q5 (Quality)	Q6 (Performance)	Q7 (Disruption)	Q8 (Options)	Q9 (Stakeholders)	Q10 (Risk)
Education	B	−.291	–	–	–	–	−.307	−.271	−.334	−.353	−.239
	t	−2.289	–	–	–	–	−3.737	−2.814	−3.583	−3.594	−2.461
	Sig.	.024	–	–	–	–	.001	.006	.001	.001	.016

Once the ANOVA tests found the statistically significant relationships between the variables, post hoc tests were conducted to find out where the significant difference is for categorical variables with three or more levels. Further, post hoc tests are used to control type 1 error (false positives). According to the conducted Levene’s Test, Tukey’s Honestly Significant Difference (HSD) was conducted when Equal Variances are assumed. The Games-Howell test was conducted when the variances among the groups are unequal. In this case, the post hoc tests were conducted only for the income source variable, as the gender variable consisted only of two levels.

Table 10 shows the Games-Howell post hoc test results for the Q8 (options) dependent variable. The test was run for the variables of Q2 (finance), Q5 (quality) and Q8 (options), where the variances among the groups are unequal. Both Q2 and Q5 results were not statistically significant. Hence, the table shows the results for Q8 only. The results show that the higher the educational level, the lower the information homeowners seek regarding the retrofit options. Tukey’s Honestly Significant Difference (HSD) was also conducted for the variables Q1, Q3, Q4, Q6, Q7, Q9 and Q10, when Equal Variances are assumed. However, all these results were statistically non-significant.

**Table 10:** Post hoc tests for income variable where the variances among the groups are unequal.

Q8 Options	Difference	Std. Error	Significance
High school < Bachelor’s degree	.738	.2202	.019
High school < Post graduate degree	1.047	.2073	.001
College < Post graduate degree	0.657	.2267	.029

## 4. Discussion

### 4.1. Summary of the findings

According to Exploratory Factor Analysis (EFA) and Cronbach’s Alpha, all the items satisfactorily contribute to the relevant information requirement code. Generally, the questions measure the construct which they are intended to measure. Although the study focused on five independent variables, statistically significant relationships were found only with education and age group variables.

**Fig 7:**
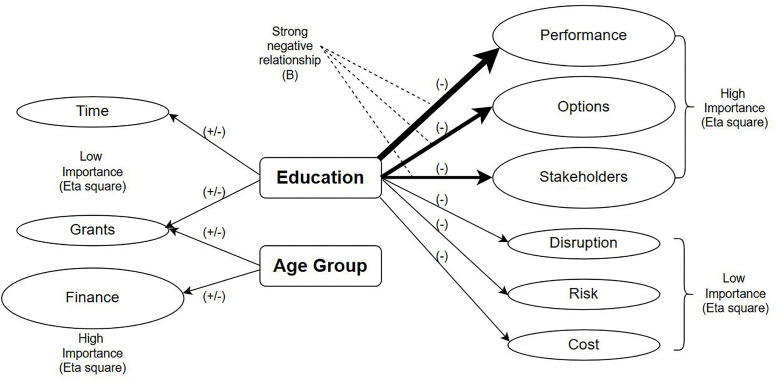
Summary of the findings.

Fig 7 shows a brief summary of the findings. Education has been identified as a key influencing factor for homeowners when they are making retrofit decisions. A strong negative relationship was found between the education independent variable and the performance, options & stakeholders dependent variables. Since this is a negative relationship, as the educational level increases, homeowners find information related to performance, options, or stakeholders are not that significant. Further, higher Partial eta square Figs were also observed with these three variables, meaning these factors have a higher practical significance.

The education variable also has a relationship with the time and grant dependent variables. However, these relationships do not show a direction of influence. Further, the age group independent variables have relationships with grants and the finance dependent variables. The direction of the relationships is unknown. However, Finance variable has a higher level of practical importance in relation to age groups.

It can be noted that the estimated energy use reductions and estimated future energy bills are the two main information items that homeowners are highly concerned about. These two items are involved with the payback of the investment and the expected utility of the retrofit. Further, the comparison of future and current energy bill/use reductions is another important information factor in data collection. This factor is also connected with the idea of payback of the retrofit investment. The sixth information factor is also related to monthly loan instalments and the payback period. It can be concluded that when homeowners look for information about retrofitting their houses, they look at housing retrofit from an investment point of view, thinking about how to recover the cost of retrofit in due course.

The third and fifth information factors are about warranty, guarantee and quality of the retrofit measures, installers and suppliers. This means homeowners focus heavily on quality to ensure that there are no detrimental effects of the retrofit process, and they need to be certain that they are getting the promised benefits without unintended consequences.

The study suggests that the homeowners’ education level clearly impacts the nature of the information they require for retrofit decision-making. Since there is a general negative relationship between the variables, it can be concluded that the higher the educational qualifications, lower the information they seek when making housing retrofit decisions. The study validates the findings of the previous literature and gives an overall idea of the homeowner decision-making behaviour in housing retrofit.

Apart from these key findings, there is a relationship between the age group of the homeowners and their decision-making behaviour under grants and finance related information. However, the direction of the relationship is inconclusive. Considering the partial eta square Figs, performance, options and stakeholders show a higher level of practical significance related to education. Due to the negative relationship, homeowners with higher educational levels do not prioritise information related to performance, options and stakeholders in the retrofit process.

### 4.2. Investment focus on housing retrofit

According to the results, homeowners see retrofit as a financial investment. Retrofit measures have unimpressive payback periods in monetary terms [24,37,38]. The energy bill savings are difficult to estimate due to reasons such as unpredictable behavioural patterns of the residents [20]. The overestimation of energy savings from retrofit measures and the need for accounting for the uncertainty were highlighted by academics. One example is the introduction of the failed 2013 Green Deal [39], and another example is the rebound effect [40].

There is also a “Prebound effect”, introduced in academia. When the residents did not use the energy as estimated for the pre-retrofit period, the retrofit would not generate the expected energy bill savings [41]. Considering these factors, it is not advisable to present the idea of “Payback” of housing retrofit with energy bill savings. This investment mentality has been infused into the homeowners by the industry itself. For example, the PAS 2035 option evaluation justifies retrofit measures according to normal payback analysis and carbon cost-effectiveness analysis [5].

It is recommended that housing retrofit be reframed as a consumption where no payback analysis is present. To reframe housing retrofit from an investment focus to a consumption focus, energy bill savings and payback period can be presented without Figs. For example, a star rating can be used to present the potential energy bill savings of a given retrofit measure. That will recommend the energy bill savings potential to the homeowner but will not push the homeowner to do payback calculations. This idea was inferred from the literature from the studies conducted by Ossokina et al and Seddiki et al [42,43].

The benefit of quality of life from retrofitting houses needs to be highlighted. Quality of life can be defined as the level of physical and mental health, wealth, comfort, necessities, and material goods available to a particular geographic area [44]. Although the financial benefits may not be attractive, retrofitting houses will improve the occupants’ health by avoiding mould growth, condensation, cold, and Volatile Organic Compounds (VOC) levels [10]. Further, it can increase thermal and indoor comfort levels [45]. Due to the potential of reducing energy bills and eliminating fuel poverty, housing retrofit shall also give financial comfort and affordability [46–48].

One in every five UK houses is reported to be below the standard quality of life [49]. 4.5 million UK houses are reported to overheat during the summer [50]. According to a study conducted by the Building Research Establishment (BRE), the direct NHS cost of poor housing is £1.4 billion for the year 2020 [51]. There are 5.6 million households in fuel poverty as of July 2024 under the 10% threshold indicator in the UK [48]. Considering the national levels, retrofit can save health costs in billions of pounds and remove nearly six million households from fuel poverty. By focusing on these reasons, the quality of life benefits of housing retrofit must be highlighted, and the investment focus needs to be reframed.

The expected utility or value of the housing retrofit should be estimated accurately, preferably without numbers but with energy efficiency potential. It will communicate retrofit benefits to the clients from the perspective of comfort, avoiding financial benefits [42]. When making decisions, humans make use of computational power, knowledge, and limited time. All of these resources are limited, and they have different levels of boundaries. This makes the concept of bounded rationality, as rationality has boundaries related to knowledge, time, and computations. In this case, people make decisions based on satisfaction, rather than perfect rationality [19,52,53]. In the context of homeowners, they are not impressed by the longer payback periods of retrofit measures, which do not satisfy their financial expectations. They will be satisfied if the retrofit benefits are presented as qualitative benefits, such as health or comfort. Further, these benefits are recommended to be aligned with their daily routine [54].

According to the literature findings and the questionnaire survey, homeowners see housing retrofit as an investment. They are looking for information on how the retrofit cost can be recovered with energy bill savings. When it comes to installing retrofit measures, homeowners look for the payback period. They do not do a payback analysis when they buy a car or install a new bathroom. Conclusively, homeowners see retrofit as an investment, but installing a new bathroom as a consumption. It is recommended to see how retrofit can be reframed to a consumption focus, away from an investment focus.

### 4.3. Unintended consequences of housing retrofit

The results have shown that the homeowners are highly concerned about the quality of the retrofit and the unintended consequences. The literature has proven that this has demotivated homeowners from engaging in the retrofit process. The earliest initiatives in retrofit can be seen after the 1970s oil crisis. Those measures included improving insulation, draught proofing, or changing the heating system [22,55]. When it comes to gas heating, they became popular after the 1960s when the North Sea gas deposits were discovered. Gas heating became very popular during the next couple of decades. Most houses were to accommodate gas central heating instead of coal [56]. There were upgrades to the doors and windows with higher energy efficiency. Double-glazing windows and higher energy-efficient doors were necessary to comply with the 2002 building regulations, subject to some exceptions [57].

The Stern Report was published in 2006, and the enthusiasm for sustainability grew as the report concluded that sustainability is cheaper in the long run [58]. The UK government published the Climate Change Act in 2008 to manage sustainable development in the country [3]. Several retrofit-related initiatives were introduced thereafter. Due to the absence of a proper quality framework, contractors without proper qualifications start installing retrofit measures to get the most out of government grants. Retrofit measures were installed without considering how different measures interact with each other. The consequences were obvious; unintended consequences occurred, such as damp, mould or structural failures. Hull, Preston and Middlesbrough’s case studies can be considered large-scale critical failures in housing retrofit [22]. People blamed the government, and the government blamed the contractors. The homeowners were the final victims. 2010–2020 has been identified as the lost decade of insulation. The statistics show that the number of insulations done in this decade is 90% lower compared to the previous decade [59]. The obvious reason could be the hesitation of the government and the fear of the homeowners.

The failed 2013 Green Deal was an eye-opener to the government about the poor quality of housing retrofit delivery [17]. Due to the increased criticisms of the quality of retrofit projects, the Each Home Counts Review was commissioned by the government and was published in 2016 by Peter Bonfield [5,21]. The recommendations were considered for adopting the PAS 2035 framework [5,23]. There are four key objectives of retrofit standards that can be identified. They shall provide clear processes to minimise risks, make retrofit a professional work, provide explicit definitions of intended outcomes with responsibility, and ensure clients’ confidence [22]. In contrast, Fylan & Glew [60] argue that the retrofit standards have not improved the retrofit quality. They further argue that the retrofit standards have increased the complexity of the installers’ work. It is expected that the installers will not be happy with the standard due to the additional accountability. Compliance with PAS 2035:2023 shall bring professionalism and responsibility into housing retrofit. It is not introduced to entertain the installers.

Apart from the PAS 2030 and 2035 specifications, several retrofit standards are observed in the context to ensure the quality of the delivery of housing retrofit. Enerphit is the certification offered to existing house retrofits under the Passivhaus scheme. The maximum heating demand for Enerphit can be below 20–30 kWh/m2a, depending on the region [61]. With references to the PAS 2030/2035 specifications and Passivhaus EnerPhit methods, now there are clear possibilities to deliver housing retrofit projects without unintended consequences.

The questionnaire survey has verified that homeowners are highly concerned about the unintended consequences of retrofit. The key reason for the fear of unintended consequences is the absence of best practices [17,22]. People without qualifications were installing single measures where there was no assessment of the risk of interactions among the measures. The risk is now mitigated to a satisfactory level with standards, certifications and best practices [62,63]. PAS 2030/2035 specifications have addressed the problem to a greater level [22]. Further, there are proven certifications such as Passivhaus EnerPhit [61]. Further, one-stop solutions for housing retrofit have the potential to improve homeowner awareness and take them through an informed retrofit process [64,65]. The existing fragmented nature of the retrofit industry is another indirect reason for the unintended consequences [66].

Although the housing retrofit has several benefits, homeowners cannot understand and interpret all these benefits when making decisions about retrofitting their houses. This can be understood in line with the previous findings of investment focus on retrofit. The homeowners seem to be focusing only on the financial outcomes of retrofitting without knowing the big picture of its importance. According to the prospect theory, people are biased between loss and gain. They show a risk-averse situation for losses and risk-seeking behaviour for gains [67,68]. This theory can be useful to explain homeowner behaviour concerning unintended consequences. Everyone is talking about the bad case study in Preston [69]. Nobody is talking about the number of successful case studies in Preston. This is the nature of good and bad news. The normal tendency is to summarise the bad and ignore the good, as explained by the “Negativity bias” [70]. The homeowners in the sample have shown risk-averse behaviour by valuing the negative information regarding the unintended consequences, as the negativity bias can be used to explain.

On the other perspective, risk-averse behaviour will be able to positively influence the homeowners to drive into housing retrofit. Li et al. (2023) suggest that a homeowner can be motivated to retrofit a house through loss framing [71]. For example, rather than talking about potential energy bill savings possible with retrofitting, talking about existing losses incurred due to not retrofitting the house can be more influential [72]. Loss framing can be interpreted under cognitive biases. The term cognitive bias was reported to have been introduced by Tversky and Kahneman in the early 1970s. According to them, the deviation patterns in human judgment are called cognitive biases. They are the wrong inferences about other people or situations, based on irrational arguments [73]. According to Ellis, when people use heuristics to make decisions without using rational judgment, they tend to make mistakes. These errors in judgment are called cognitive biases [74]. It is recommended to explore ways to use cognitive biases to positively engage homeowners in the housing retrofit process.

### 4.4. Level of education of homeowners

The questionnaire survey showed that the decision-making behaviour of the homeowners is sensitive to their level of education. Regarding the demographics of the sample, 75% of respondents have a bachelor’s degree or a master’s degree. Considering the demographics of England and Wales, only around 30% of the population has a higher educational qualification. The study has a limitation: the sample is not representative of the population in England and Wales in terms of education. It is important to understand that the findings will be mainly applicable to people with higher educational qualifications. On the other point, the finding is related to the level of education, and it will not have a considerable impact on the other findings.

**Fig 8:**
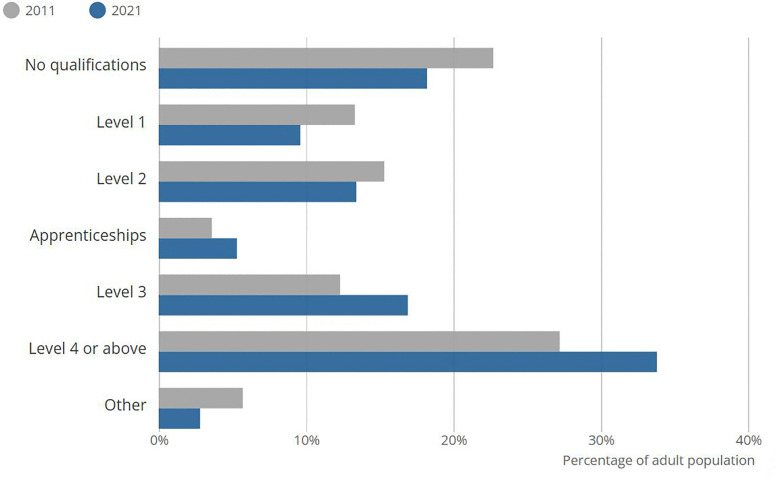
Highest level of qualification in England and Wales 2011–2021 [75].

Fig 8 shows the educational qualification levels of the residents in England and Wales. UK residents are not observed to be aware of energy efficiency. A study conducted by Citizens Advice UK has found that 73% of homeowners do not know about their energy performance rating. This study highlights the importance of homeowner awareness about the benefits of energy efficiency [76]. This corresponds with the data from Fig 8 about the educational levels. Nearly 70% of the population does not have a higher education. Having noted that, it is still impossible to conclude that people without higher education are not aware of energy efficiency.

It was observed that people find it difficult to be convinced to do things even if they are beneficial to them. For example, some people chose not to get vaccinated during the COVID-19 pandemic even though that was a matter of life and death [77]. Awareness is important, but it is not everything. Under practice theories, people make decisions that they are doing as a routine [78]. Considering these aspects, there is a timely need to make people aware of the benefits of retrofit as well as understand their decision-making to influence behavioural changes [79–81]. Awareness of the residents will be the first priority in the retrofit delivery. Even if they choose to retrofit or not, the journey will start with awareness and proceed to the next stages.

Engaging homeowners in the retrofit process has been identified as a key challenge in driving retrofit [82,83]. With the understanding of the sensitivity of homeowners’ educational level to housing retrofit decision-making, it is important to evaluate how this finding can be put into practice. It can be concluded that the different homeowners with different levels of education, need to be approached differently. According to the results, homeowners with lower educational levels will seek more information when making retrofit decisions. This highlights the need for better and more awareness-raising programmes since 70% of the population do not have higher educational qualifications.

The message of retrofit needs to be tailored according to the level of education and capacity of the homeowner. This idea aligns with the recommendations of constructivist approaches to propose socio-cultural dimensions instead of technical ones [84,85]. Considering the first finding related to the homeowner’s attitude towards retrofit being viewed as an investment, Massironi et al [86] discuss investment decision-making based on the decision-maker’s “constructivist awareness” based on their own constructs and cognitive processes.

Besides the educational levels of the homeowners, learning theories can be used to engage homeowners. According to Jerome Bruner’s theory of spiral curriculum, the learning content should be presented to the learner in iterations, each iteration giving the learner more complex knowledge [87]. This is also in line with the cognitive load theory, which recommends adapting learning content to the learner’s capacity [88]. By adapting this to the homeowner decision-making behaviour, homeowners should be engaged in the retrofit process in an iterative manner while adapting to their capacity. It shows a need to focus on the homeowner’s socio-cultural and demographical background to disseminate the idea of housing retrofit.

## 5. Conclusion

The study looked into the homeowner behaviour in housing retrofit decision-making by way of a questionnaire survey, which focused on the homeowner demographics; age group, education, income level, income source and gender. The findings were derived from the descriptive statistics and ANOVA test, which showed satisfactory statistical significance. The results can be mainly generalised for the UK population with some level of higher educational qualifications and who are employed. Despite this limitation, the study has signposted valuable findings related to homeowner behaviour.

The first conclusion of the study is that the homeowners look at housing retrofit from an investment point of view. Homeowners are demotivated to retrofit their houses, as most of the housing retrofits do not have a satisfactory payback in financial terms. It is recommended to reframe housing retrofit as a consumption, not an investment. The homeowners are highly concerned about unintended consequences when making a housing retrofit decision. In this case, it is critically important to build the confidence of the homeowners through standards, best practices, research and innovation.

The ANOVA test found that the homeowners’ educational level is related to the information they seek when making a retrofit decision. Homeowners with lower educational levels seek more information when making a retrofit decision. Since 70% of residents of England and Wales do not have higher educational qualifications, it is recommended to increase awareness related to retrofit, in order to engage them more in the housing retrofit process.

Despite these findings, the study has limitations as the sample mainly consists of highly educated and employed homeowners. Further studies are recommended to address these limitations.
